# Global research trends in neurosyphilis: a bibliometric analysis from 2010 to 2024

**DOI:** 10.3389/fimmu.2025.1649106

**Published:** 2025-10-02

**Authors:** Wei Wei, Wei Li

**Affiliations:** ^1^ Department of Clinical Laboratory, The Affiliated Hospital of Jiaxing University, Jiaxing, China; ^2^ Jiaxing Key Laboratory of Clinical Laboratory Diagnosis and Transformation Research, The Affiliated Hospital of Jiaxing University, Jiaxing, China

**Keywords:** neurosyphilis, bibliometric analysis, *Treponema pallidum*, cerebrospinal fluid, asymptomatic neurosyphilis

## Abstract

**Background:**

Neurosyphilis, as a serious infectious disease caused by *Treponema pallidum* invading the central nervous system, has seen a significant increase in global incidence in recent years. However, the trends and gaps in the research of neurosyphilis remain unknown.

**Objective:**

Bibliometrics was adopted to analyze the research trends of neurosyphilis from 2010 to 2024, and to identify the core themes, hotspots and development directions.

**Methods:**

Research related to neurosyphilis from 2010 to 2024 was retrieved in the Web of Science core Collection (WOSCC). Bibliometrix, VOSviewer, CiteSpace, and BioBERT language models were employed to perform bibliometric and knowledge mapping analyses on global research output, author/institution collaboration networks, keyword evolution, co-citation clusters, and associated genes in the field of neurosyphilis.

**Results:**

A total of 863 articles were included in the analysis. From 2010 to 2024, both the number of publications and citations demonstrated a rapid upward trend. The United States and China were the leading contributors in the field of neurosyphilis research, accounting for 27.3% and 26.9% of global publications, respectively. The University of Washington and Xiamen University emerged as the most prolific research institutions. Keyword analysis identified “ cerebrospinal fluid (CSF),” “ human immunodeficiency virus (HIV),” “ocular syphilis,” and “general paresis” as core research topics. Research focus has gradually shifted from traditional diagnostic criteria and penicillin-based treatment approaches to investigations into immune mechanisms, co-infection factors, and the identification of novel biomarkers. In recent years, the appearance of keywords such as “case report,” “gene expression,” and “transcriptomics” indicates a growing emphasis on precision medicine and molecular mechanisms. The increasing frequency of immune-related molecules, including CD4, CXCL13, and IL-6, suggests that the mechanisms underlying immune responses may represent a promising direction for future research breakthroughs.

**Conclusions:**

Research on neurosyphilis is transitioning from traditional clinical descriptions toward multidisciplinary precision medicine. Future efforts should focus on integrating multi-omics technologies, establishing globally unified diagnostic criteria, and enhancing international collaboration to address the public health challenges posed by this disease.

## Introduction

Neurosyphilis is a chronic infectious disease caused by the invasion of *Treponema pallidum* into the central nervous system, resulting in meningeal, vascular, and parenchymal damage, and a wide range of clinical manifestations including cognitive impairment, movement disorders, and psychiatric symptom (1, 2). Although the widespread use of penicillin led to a period of “silence” for syphilis in the 1990s, the disease has experienced a global “resurgence” in recent years, with an increasingly severe epidemiological situation (3–5). Concurrent with this resurgence, the incidence of neurosyphilis has also risen. According to the Centers for Disease Control and Prevention (CDC), the number of neurosyphilis cases increased by 32% between 2020 and 2021 (6). Currently, the diagnosis of neurosyphilis relies on CSF analysis, serologic tests, and imaging techniques. However, the sensitivity and specificity of these methods remain controversial, and there is a lack of global standardization in diagnostic criteria (7). In recent years, the exploration of CSF biomarkers (e.g., CXCL13, interleukin-6), advances in molecular diagnostic techniques [e.g., polymerase chain reaction (PCR), metagenomic sequencing], and novel imaging modalities have begun to reshape traditional diagnostic and treatment approaches (8–11). However, existing studies are scattered across fields such as epidemiology, molecular biology, neuroimaging, and clinical therapeutics, lacking systematic integration. This fragmentation makes it difficult to clearly understand the evolution and future direction of neurosyphilis research. Bibliometric analysis offers an objective method to reveal the knowledge structure and developmental trends of a research field by quantitatively analyzing literature output, author collaboration networks, keyword hotspots, and research frontiers. It has been widely applied in the study of neurological diseases such as Alzheimer’s disease and multiple sclerosis (12, 13), yet no such systematic analysis exists for neurosyphilis. This gap has limited researchers’ understanding of core academic contributors, interdisciplinary potential, and the application of emerging technologies, thereby hindering knowledge dissemination and innovation. Therefore, this study adopted a bibliometric approach using data from the WOSCC database (2010–2024) to identify key research themes, hotspots, and future directions, thereby supporting clinical decision-making, guiding research planning, and informing policy development.

## Materials and methods

### Data sources

The data for this study were obtained from the WOSCC database (https://www.webofscience.com/wos/woscc/basic-search). WOSCC was selected for its widespread use in bibliometric studies and its rigorous inclusion criteria, ensuring the authority and representativeness of the literature (14).

### Search strategy

The search formula was set to [TS = (neurosyphilis) or (Syphilis, General Paralysis) or (syphilis, central nervous system) or (Syphilis, General Paresis) or (Syphilis, Juvenile Paresis) or (Syphilis, Tabes dorsalis)] and LA = (English). The type of documents was set to “articles” with a timespan ranging from January 1, 2010 to December 31, 2024. Studies not related to neurosyphilis were excluded according to the title and abstract. Studies that had been withdrawn were excluded.

### Data analysis tools

Bibliometric analysis was performed using the Bibliometrix R package (version 4.3.0) to examine global research patterns related to neurosyphilis, including key journals, authors, citations, keywords, institutions, countries, and co-occurrence networks (15). VOSviewer (version 1.6.20) was used to construct collaboration networks between countries and institutions, and to generate keyword co-occurrence maps (16). CiteSpace (version 6.3.R1) was used to identify highly cited keywords and to cluster co-citations for thematic analysis (17). BioBERT trains domain-specific language models using large-scale biomedical datasets (18). It is first initialized with BERT weights and pre-trained on public corpora. Subsequently, the pre-trained model is further optimized using biomedical corpora.

## Results

### Annual publication trends

**Figure 1:**
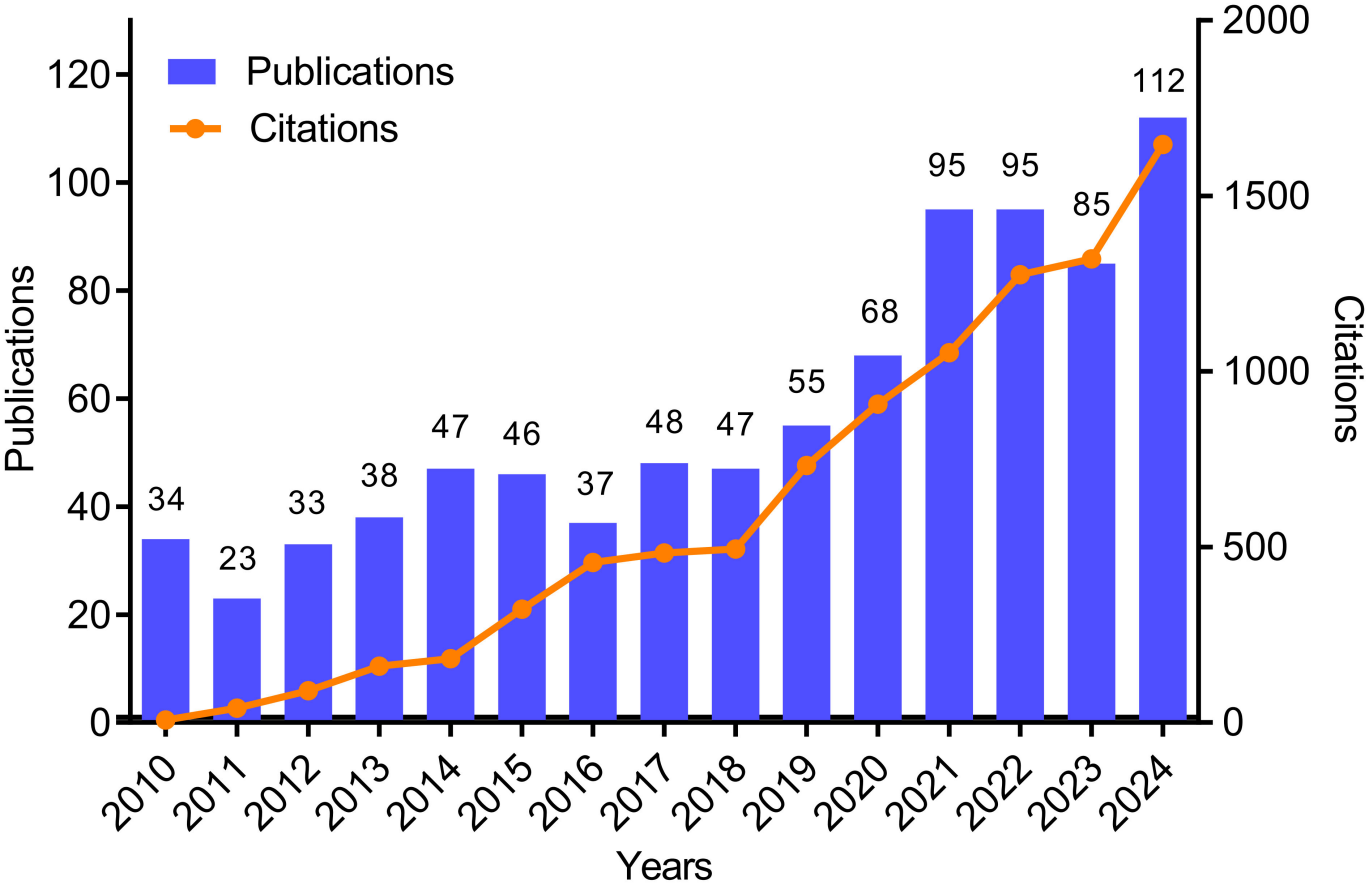
Annual number of publications and total annual citations for neurosyphilis-related research. Bar graph shows the annual number of publications; line graph shows the total annual citations.

From 2010 to 2024, a total of 863 publications related to neurosyphilis were obtained. The total number of annual publications and citations are presented in **Figure 1**. In 2010, there were approximately 34 publications related to neurosyphilis. By 2011, the number of publications had sharply declined to about 23; however, the total annual citation count increased compared to 2010. Between 2011 and 2016, the number of publications initially increased and subsequently decreased, reaching a minor peak of 47 publications in 2014. From 2017 to 2022, the number of publications steadily increased. In 2023, the number of publications significantly declined to 85, followed by a peak of 112 publications in 2024. The total annual citation count exhibited a gradual upward trend from 2010 to 2016, entered a plateau phase between 2017 and 2018, and experienced a rapid increase from 2019 to 2024. Overall, both the number of publications and citations demonstrated a rapid growth trend from 2010 to 2024, indicating a significant increase in research activity and the expanding influence of scholarly outputs in this field.

### Author country/region distribution

**Figure 2:**
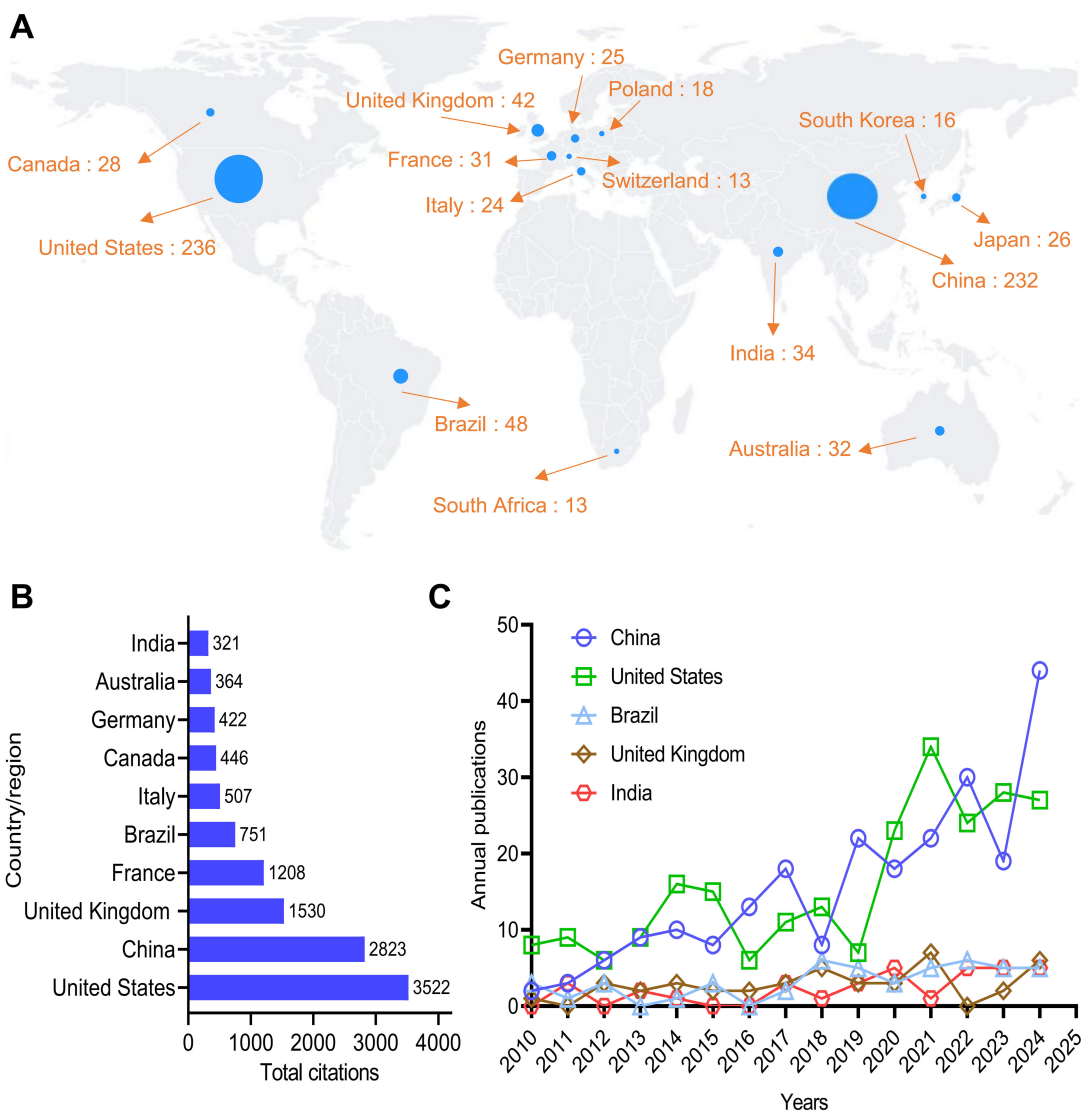
Distribution of authors by country/region. **(A)** Map of the distribution of authors. **(B)** Top 10 countries by total citations. **(C)** Publication trends over time for the top five countries.

The top 15 countries or regions in the field of neurosyphilis research from 2010 to 2024 are presented in **Figure 2A**. The United States of America contributed the highest number of publications in this field, with 236 articles (27.34%), followed by China with 232 articles (26.88%). Brazil ranked third with 48 articles (5.56%), followed by the United Kingdom with 42 articles (4.86%), and India with 34 articles (3.93%). France occupied the sixth position, contributing 31 articles (3.59%). **Figure 2B** illustrates the top 10 countries in terms of total citation counts. Publications from the United States received the highest cumulative citations, totaling 3,522, followed by China with 2,823 citations. **Figure 2C** depicts the temporal evolution of publication outputs from the top five countries. All five countries demonstrated an upward trend over time. The annual number of publications from China and the United States was comparable and exhibited rapid growth. In contrast, Brazil, the United Kingdom, and India experienced slower growth, although the increase was less pronounced compared to China and the United States. These findings suggest a growing global interest in neurosyphilis research.

### Contribution of publishing institutions

**Figure 3:**
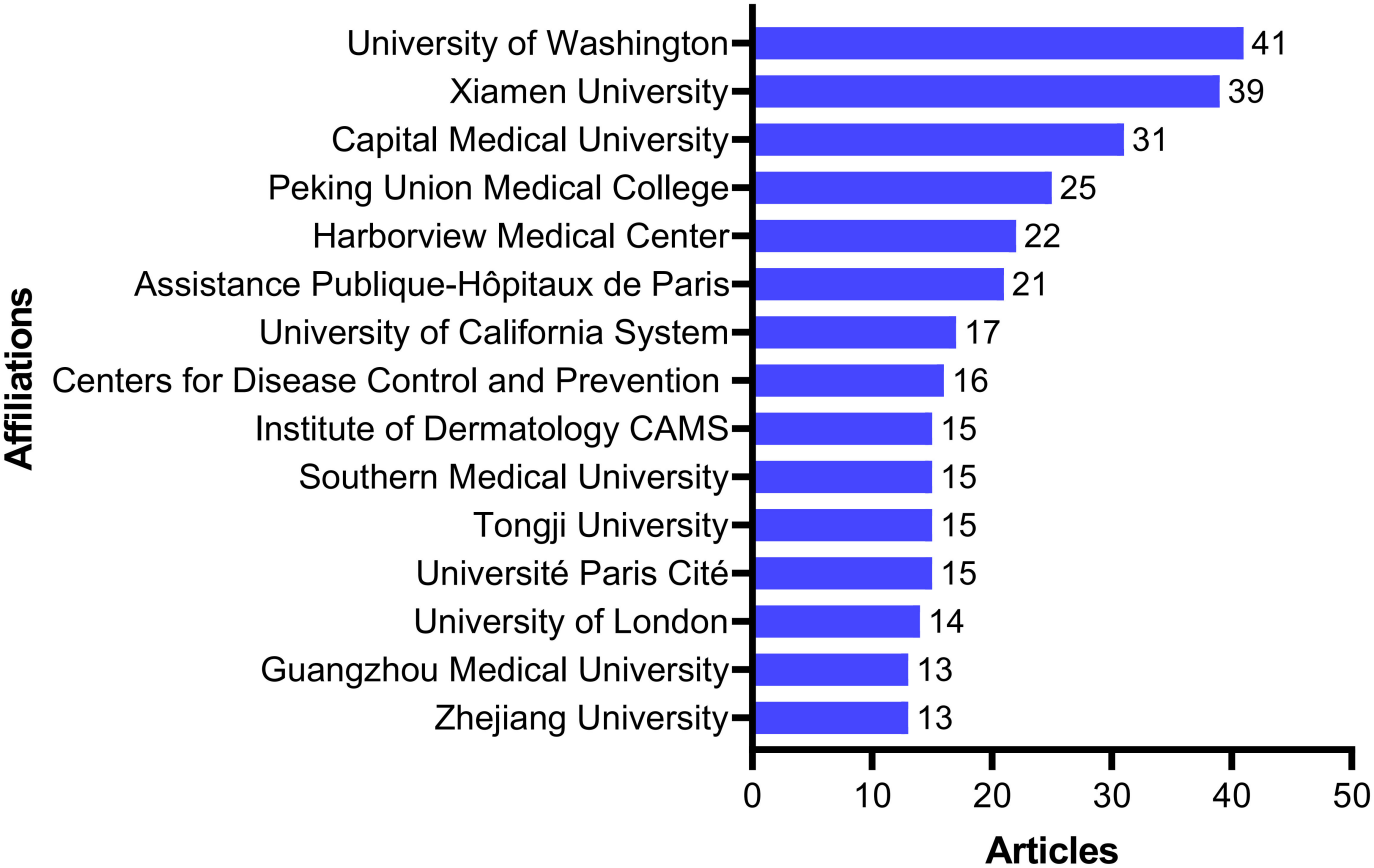
The top 15 institutions in the number of publications.

Understanding publication trends across institutions can provide insights into shifts in research activity and scholarly output. **Figure 3** presents the 15 institutions with the highest number of publications globally. Among them, 8 are from China, 4 from the United States, 2 from France, and 1 from the United Kingdom. The University of Washington ranked first with 41 publications, followed by Xiamen University with 39 publications. Other leading institutions include Capital Medical University (31 publications), Peking Union Medical College (25 publications), Harborview Medical Center (22 publications), and Assistance Publique-Hôpitaux de Paris (21 publications).

### Analysis of publishing journals

**Figure 4:**
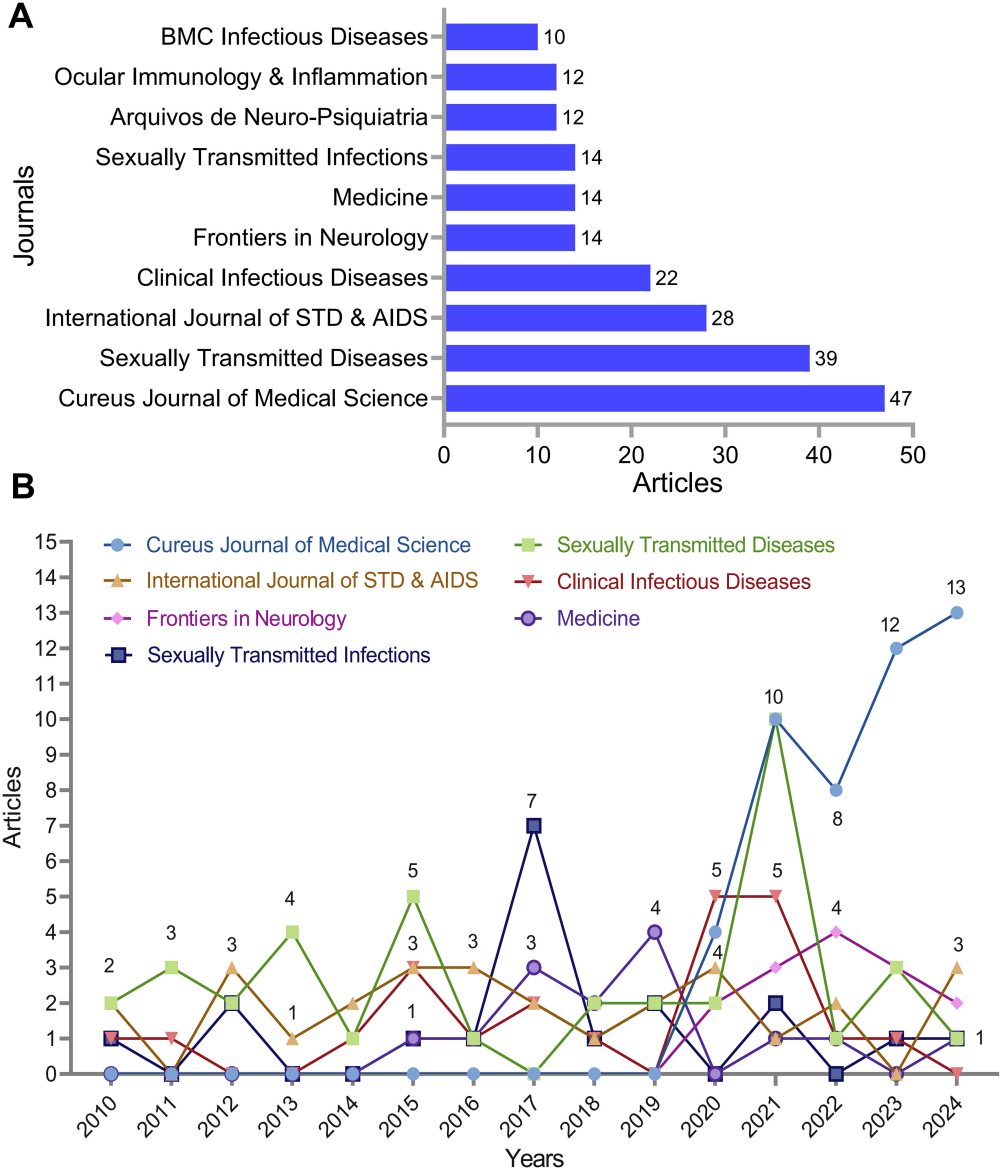
Analysis of journals with neurosyphilis publications. **(A)** Top 10 journals by publication count. **(B)** Publication trends over time for the top 7 journals.

The top 10 journals publishing neurosyphilis-related articles from 2010 to 2024 are shown in **Figure 4A**. *Cureus Joumnal of Medical Science* has the most publications (n = 47), followed by *Sexually Transmitted Infections* (n = 39), and *International Joumal of STD & AIDS* (n = 28). Among these journals, *Cureus Journal of Medical Science* emphasizes the publication of case reports. *Medicine* publishes research across a broad spectrum of medical science disciplines and subspecialties, while the remaining 8 journals primarily focus on research in infectious diseases and neuroscience. Data indicate a consistent rise in neurosyphilis-related publications in the top 6 journals (**Figure 4B**), suggesting sustained research efforts and the increasing significance of this field.

### Study authors and cooperation between authors

**Figure 5:**
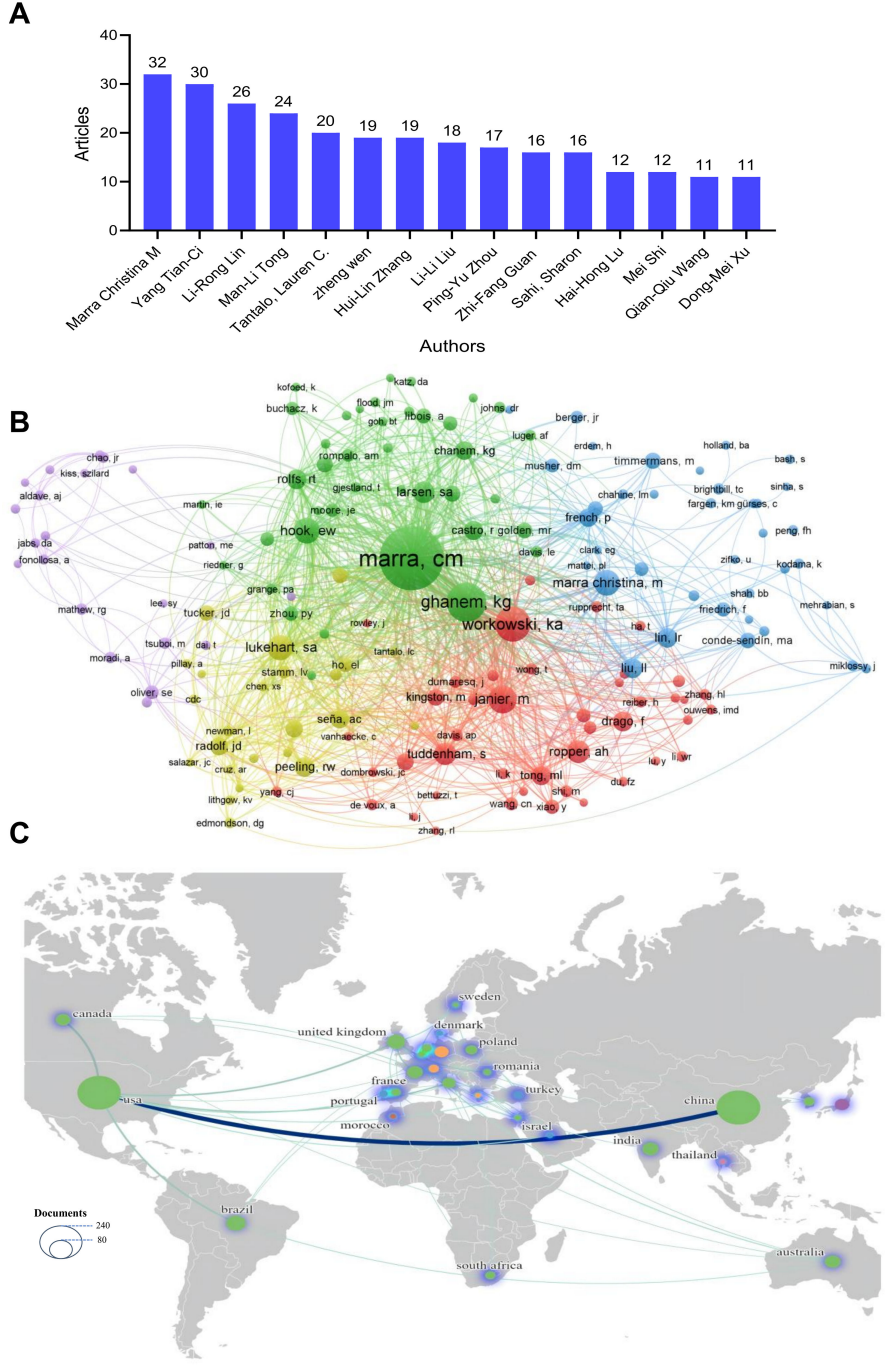
Authors and collaboration analysis. **(A)** Top 15 authors ranked by the number of publications. **(B)** Author collaboration network map. **(C)** Country collaboration map. The size of the circle indicates the number of articles published. Thicker lines indicate stronger cooperation.

The top 15 authors in the field of neurosyphilis published globally from 2010 to 2024 are shown in **Figure 5A**. Among these 15 authors, two are affiliated with the United States, while the remaining authors are all from China. Marra Christina M is the most prolific contributor in this field, having published a total of 32 articles, followed by Yang Tian-Ci with 30 publications. Subsequently, Li-Rong Lin (n=26), Man-Li Tong (n=24), and Tantalo Lauren C (n=20) also made significant contributions. Collaboration and communication among these authors facilitate the formation of academic networks, enhance the dissemination of relevant knowledge, and promote scholarly exchange. **Figure 5B** illustrates the co-authorship network among the authors, which has been clustered into five distinct groups. As the most influential researcher in the field, Marra Christina M exhibits high values in both mediation centrality and PageRank, underscoring her pivotal role within the research community. Other researchers, such as Ghanem KG and Workowski KA, although less prominent than Marra Christina M in neurosyphilis research, still hold notable positions within the co-authorship network, indicating their involvement and influence. **Figure 5C** presents a global map of international collaboration. The most frequent collaboration occurs between China and the United States. Additionally, European countries, including the United Kingdom, Italy, Poland, and France, demonstrate a strong tendency toward collaborative research. Overall, the map reveals limited global collaboration, highlighting the specialized and relatively independent nature of research in this domain.

### Citation analysis of publications

**Figure 6:**
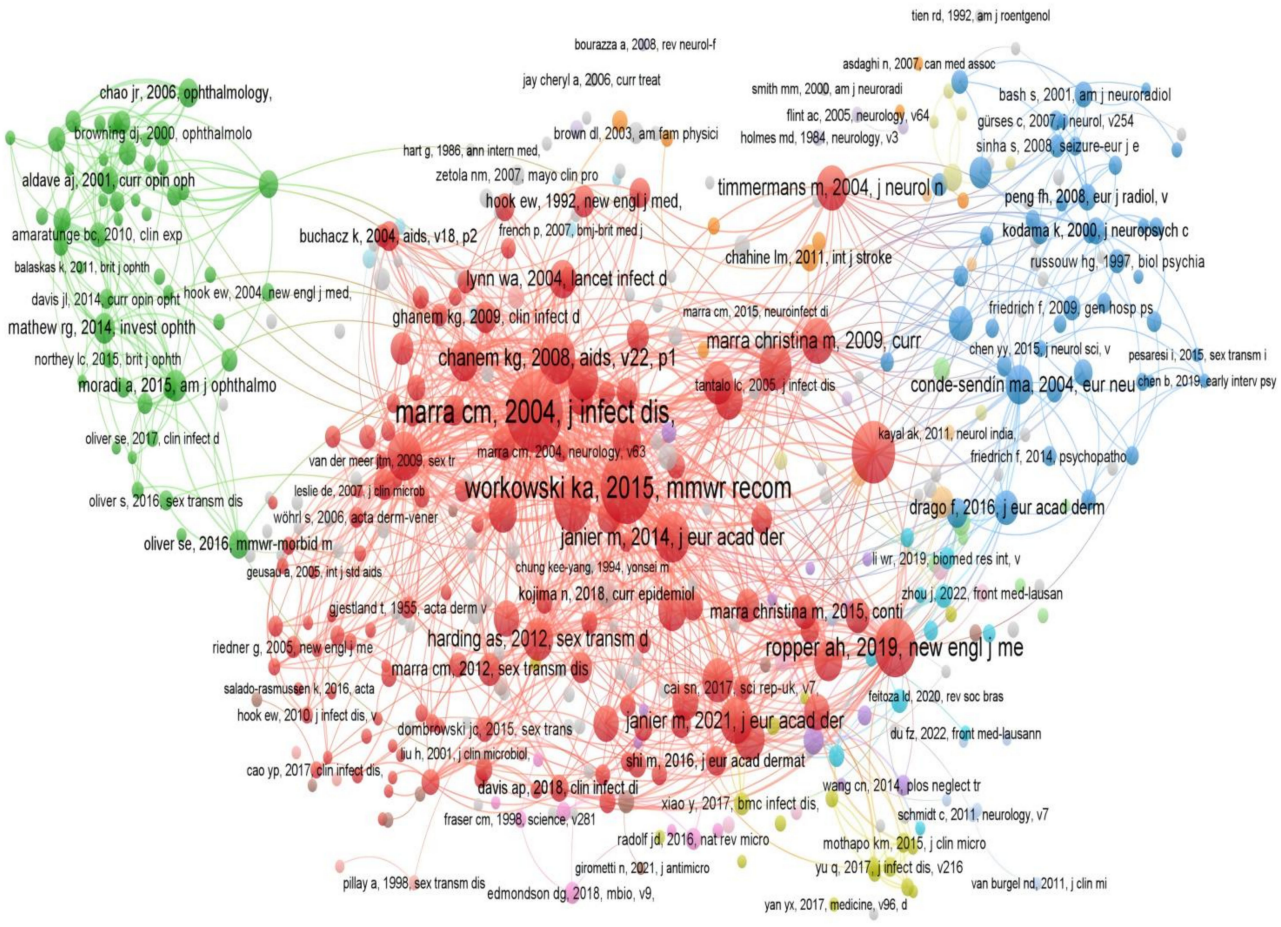
Literature co-citation analysis of neurosyphilis publications.

Citation status can reflect the quality and importance of a study, and frequently cited literature often represents the research hotspots and high points in a certain field. Long-term tracking of the citation of a discipline can reveal the development course, current situation and future trend of the discipline. The top 15 cited publications in the field of neurosyphilis from 2010 to 2024 are shown in **Table 1**. The most cited article was published by Peeling RW in 2017, which received 404 citations. This was followed by the articles published by Janier M in 2014 and 2017, which were cited 278 and 227 times, respectively. These publications focused on research topics such as ocular syphilis, molecular typing of syphilis, diagnostic methods, and treatment guidelines. Literature co-citation analysis was further conducted to identify influential studies or research groups in the field and the interrelationship between research topics. As shown in **Figure 6**, the network diagram shows that Marra CM published the most influential literature in *Journal of Infectious Diseases* in 2004. Its high mediation centrality and PageRank value highlighted its central role in the research community. In addition, other literature such as Workowski KA 2015 and Ghanem KG 2008 have shown their importance in neurosyphilis research. The scarcity of post-2010 nodes in the central cluster indicates that recent investigations have largely validated earlier findings rather than introduced paradigm-shifting discoveries.

**Table 1:** Top 15 publications ranked by total citations.

Ref.	Year	Title	Total citations
Peeling RW et al. (68)	2017	Syphilis	404
Janier M et al. (69)	2014	2014 European guideline on the management of syphilis	278
Janier M et al. (70)	2021	2020 European guideline on the management of syphilis	227
Kingston M et al. (71)	2016	UK national guidelines on the management of syphilis 2015	175
Marra CM et al. (60)	2010	Enhanced Molecular Typing of Treponema pallidum: Geographical Distribution of Strain Types and Association with Neurosyphilis	157
Eandi CM et al. (72)	2012	Acute syphilitic posterior placoid chorioretinitis: report of a case series and comprehensive review of the literature.	133
Pichi F et al. (73)	2014	Spectral domain optical coherence tomography findings in patients with acute syphilitic posterior placoid chorioretinopathy	113
Lepennetier G et al. (52)	2019	Cytokine and immune cell profiling in the cerebrospinal fluid of patients with neuro-inflammatory diseases	111
Oliver SE et al. (74)	2016	Ocular Syphilis - Eight Jurisdictions, United States, 2014-2015	105
Grange PA et al. (75)	2012	Evaluation of a PCR test for detection of treponema pallidum in swabs and blood	102
Marra CM et al. (9)	2010	CXCL13 as a cerebrospinal fluid marker for neurosyphilis in HIV-infected patients with syphilis	97
Dutta Majumder P et al. (76)	2019	Ocular Syphilis: An Update	87
Hytönen J et al. (77)	2014	CXCL13 and neopterin concentrations in cerebrospinal fluid of patients with Lyme neuroborreliosis and other diseases that cause neuroinflammation	80
Mattei PL et al. (78)	2012	Syphilis: A Reemerging Infection	79
Bissessor M et al. (79)	2010	Frequent Screening for Syphilis as Part of HIV Monitoring Increases the Detection of Early Asymptomatic Syphilis Among HIV-Positive Homosexual Men	73

### Analysis of research hotspots

**Figure 7:**
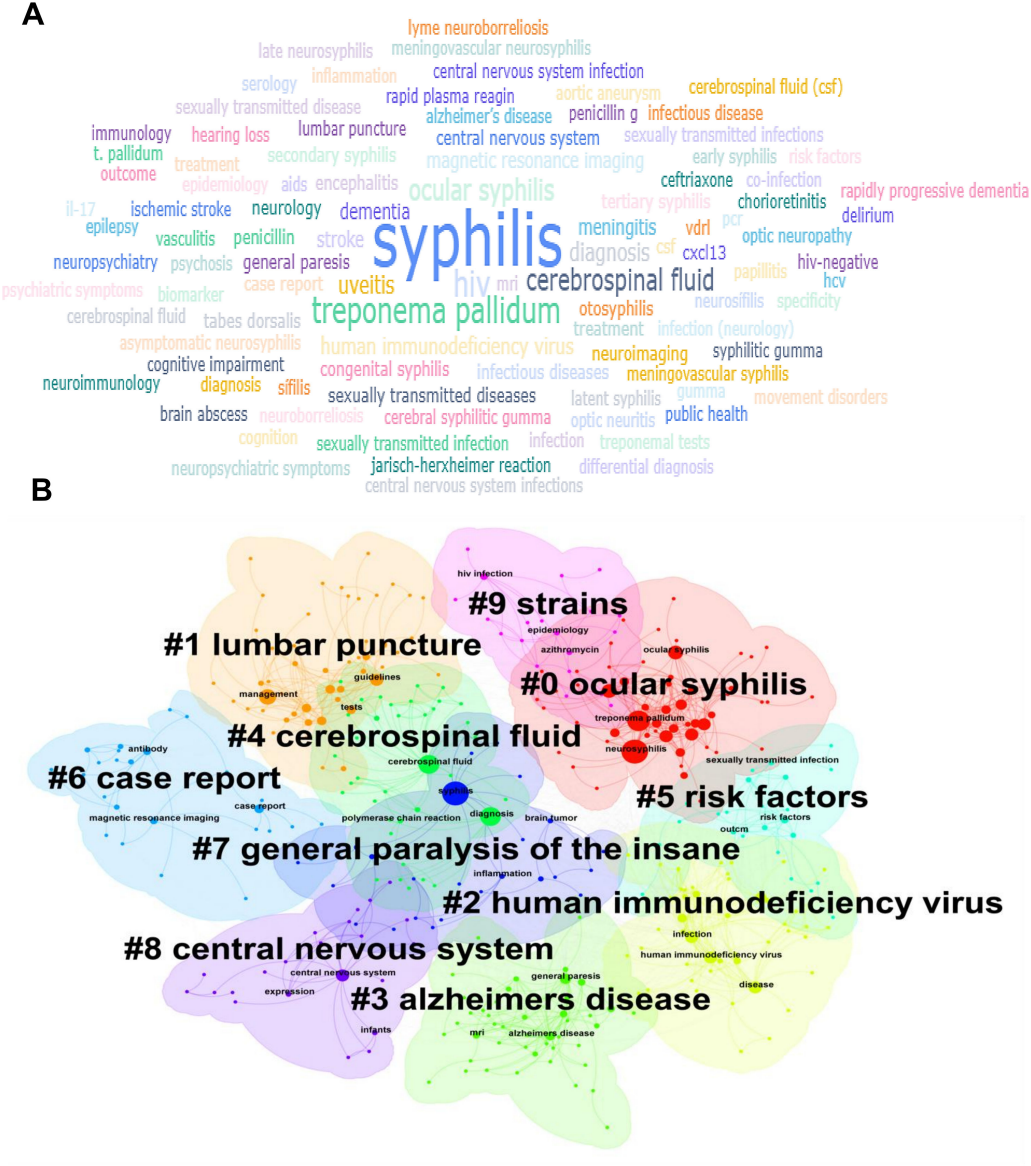
Publication keyword co-occurrence analysis. **(A)** Keyword co-occurrence map for neurosyphilis research. Larger fonts indicate higher keyword frequencies. **(B)** Cluster analysis of keywords.

The key words of the thesis are the purpose, object and method of the research. The analysis of keywords can reflect the trend of topic evolution and research hotspots in a certain research field during a certain period of time. We first analyzed the frequency of keywords related to the field of neurosyphilis. As shown in **Figure 7A**, the top 5 keywords with the highest frequency were syphilis, CSF, *Treponema pallidum*, HIV, and ocular syphilis. The keywords were analyzed by cluster analysis (**Figure 7B**). These keywords can be divided into 10 distinct significant clusters, which are: ocular syphilis (#0), lumbar puncture (#1), HIV (#2), Alzheimer’s disease (#3), CSF (#4), risk factors (#5), case report (#6), general paralysis of the insane (#7), central nervous system (#8), strains (#9). The three clusters of CSF (#4), central nervous system (#8) and lumbar puncture (#1) all pointed to the diagnostic process of central nervous system infection, suggesting that a standardized research framework with CSF assessment as the core has been formed in this field. The co-occurrence of three clusters, HIV (#2), risk factors (# 5) and strains (# 9), indicated that HIV co-infection was the main engine driving the case discovery and research output of neurosyphilis. ocular syphilis (#0) and general paralysis of the insane (#7) occupy a place in the high-frequency words, indicating that ocular syphilis and classic paralysis of the insane (#7) have become research hotspots again, and tend to be independent subtypes. case report (#6) clustering showed that the current evidence level was still dominated by case series, which was the main way to generate new phenotype description and diagnostic clues. The Alzheimer’s disease (#3) cluster suggests that neurosyphilis is being included in the differential diagnosis of “reversible dementia/young dementia”, forming an intersection with the field of neurodegenerative diseases. Q-values and S-values are metrics to describe network structure and clustering. The results of this study were Q = 0.4953 and S = 0.8032, which met the study criteria. Therefore, the clustering results are reliable for subsequent analysis.

**Figure 8:**
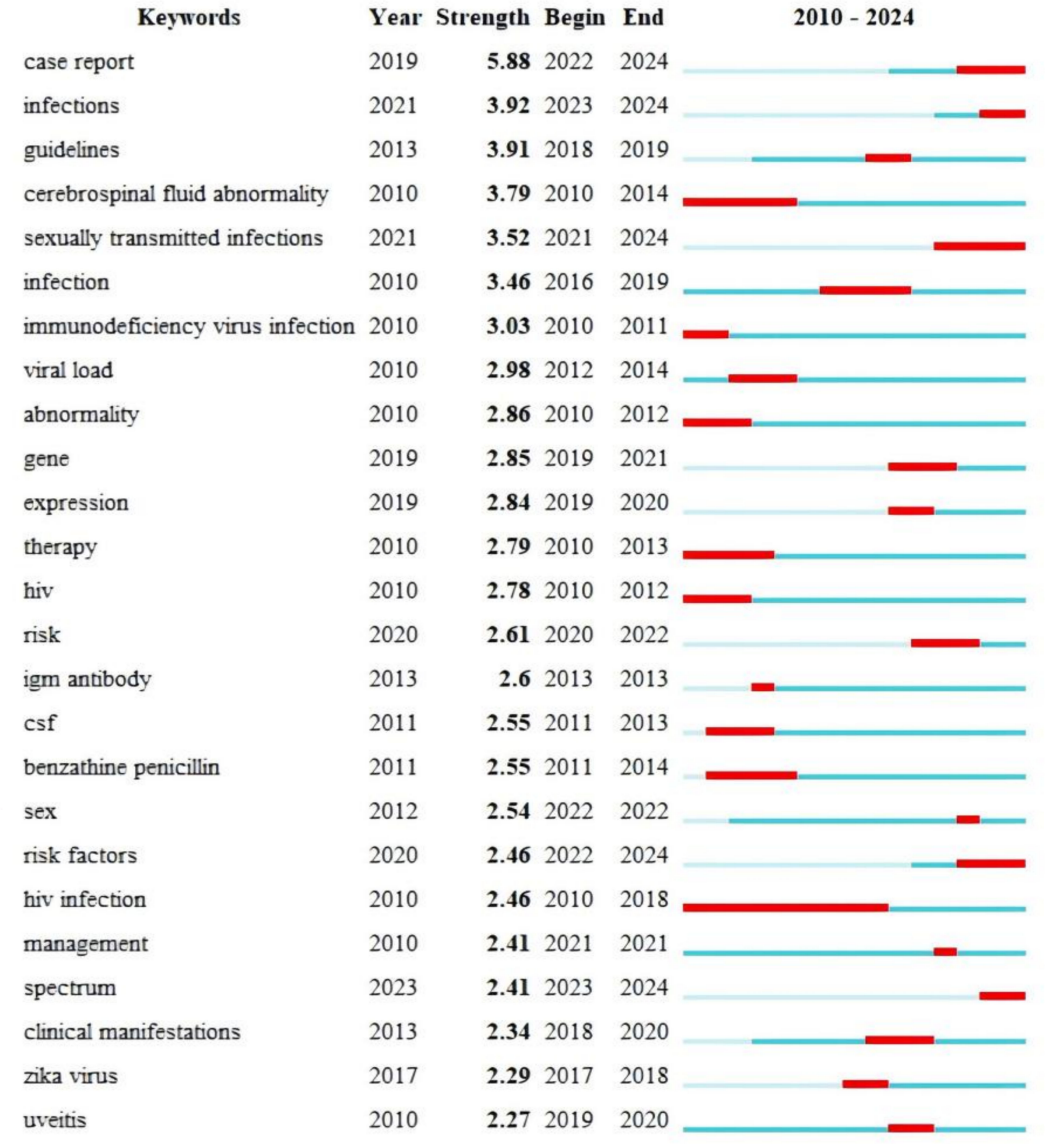
Keyword breakout analysis.

Keyword breakout analysis reveals the evolution and directional shifts of research hotspots by identifying keywords that exhibit sudden increases in frequency or strength within a short time interval. The burst keyword sequence of neurosyphilis-related literature from 2010 to 2024, which can be broadly categorized into three developmental stages (**Figure 8**). The first phase (2010-2014) was characterized by emergent keywords such as “cerebrospinal fluid abnormality”, “viral load”, “HIV”, “therapy”, “CSF”, and “benzathine penicillin”. These findings indicate that this stage primarily focused on establishing CSF evaluation criteria and penicillin-based treatment protocols in the context of HIV co-infection, thereby laying a methodological foundation for subsequent studies. The second phase (2015-2020) witnessed the emergence of keywords such as “guidelines”, “clinical manifestations”, “infection”, and “uveitis”, reflecting the publication and citation of clinical management guidelines for neurosyphilis. During this period, research on ocular syphilis was introduced, and the disease phenotype was further refined. The third phase (2021-2024) was marked by the prominence of “case report”, “sexually transmitted infections”, and “risk factors”. Notably, the burst intensity of “case report” reached 5.88, suggesting that rare clinical manifestations were increasingly documented through case reports. Additionally, the transient appearance of “gene” and “expression” between 2019 and 2021 indicates the initial emergence of research on transcriptomics and CSF cytokines, although this area has not yet become a sustained mainstream focus.

### Analysis of associated genes

**Figure 9:**
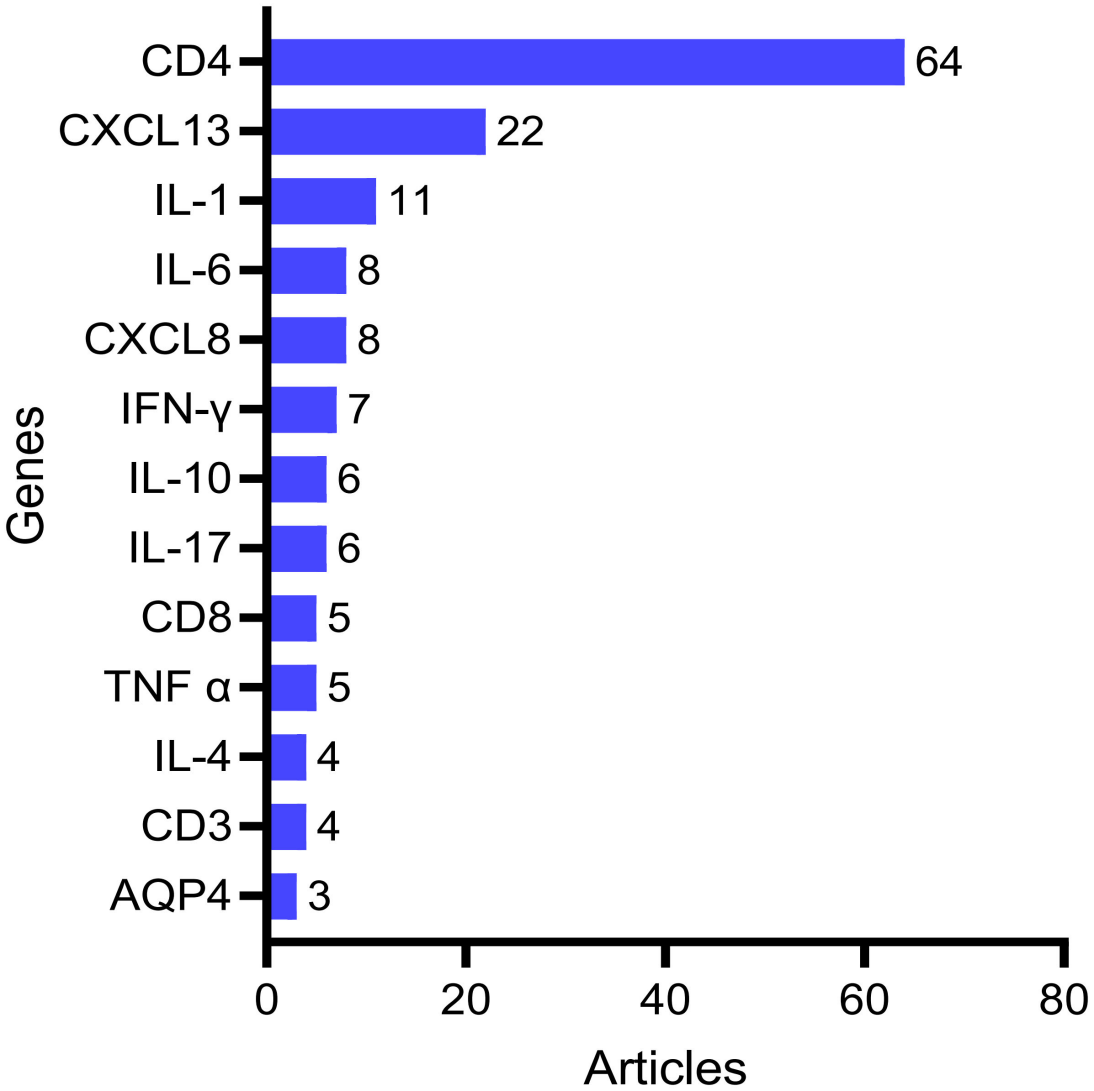
Analysis of associated genes.

The BioBERT biomedical language representation model was employed to extract and statistically analyze gene/molecule entity terms from the abstracts of 862 publications. As illustrated in **Figure 9**, the top 13 genes/molecules identified are as follows: CD4 (64 articles), CXCL13 (22 articles), IL-1β (11 articles), IL-6 (8 articles), CXCL8 (8 articles), IFN-γ (7 articles), IL-10 (6 articles), IL-17 (6 articles), CD8 (5 articles), TNF-α (5 articles), CD3 (4 articles) and AQP4 (3 articles).

## Discussion

This study conducted a systematic analysis of global research trends in the field of neurosyphilis from 2010 to 2024 using bibliometric methods. The findings highlight significant advancements in diagnostic techniques, epidemiological characteristics, and molecular mechanisms within the field, while also outlining existing challenges and potential directions for future research.

### The evolution of diagnostic methods for neurosyphilis

This study found that research on neurosyphilis diagnosis has transitioned from traditional serological methods to the use of diverse biomarkers. Early studies primarily focused on establishing CSF assessment criteria and optimizing penicillin treatment regimens, closely tied to the rising prevalence of HIV co-infection at the time (19, 20). Notably, the study by de Almeida SM et al. (21) further confirms that CSF white blood cell counts and total protein levels exhibit significant variations in diagnostic performance across different populations, particularly limiting the applicability of these traditional biomarkers in HIV-infected individuals. Multiple studies have demonstrated that CXCL13 had excellent sensitivity and specificity in the diagnosis of neurosyphilis (22–24). The significance of CXCL13 in CSF as a diagnostic marker for neurosyphilis lies in its ability to directly quantify B cell chemotactic activity within the central nervous system with high sensitivity. The systematic review demonstrated that the pooled sensitivity was 0.76, specificity was 0.83, and the AUC was 0.84, with notably improved performance observed in the Chinese population and the HIV-negative subgroup (25). This finding may facilitate the early identification of nerve invasion during the CSF-VDRL negative or asymptomatic stages, thereby reducing the diagnostic window. In addition, CXCL13 levels are positively correlated with white blood cell count, total protein concentration, and TPPA titer in CSF (24). Moreover, CXCL13 levels are significantly reduced following treatment for neurosyphilis (23). These findings suggest that CXCL13 serves as a valuable biomarker for monitoring therapeutic efficacy and predicting disease recurrence, thereby providing a quantifiable and dynamic indicator for incorporating “immune clearance” into the assessment of treatment outcomes. However, existing studies exhibit significant variability in threshold values, with HIV co-infection, sample size, and regional heterogeneity all impacting their stability. Future multi-center, prospective trials are needed to validate a unified cutoff point and explore algorithms combining it with CSF-WBC and TPPA. This will facilitate the transition of CXCL13 from a laboratory marker to a routine clinical tool. In recent years, the application of PCR technology in the diagnosis of neurosyphilis has advanced significantly, establishing it as an important complementary tool to traditional serological and CSF examination methods. Studies have demonstrated that PCR assays targeting specific genes of *Treponema pallidum* (e.g., tpp47 and polA) exhibit high diagnostic specificity in CSF samples (26, 27), particularly in cases presenting with indeterminate serological findings or atypical clinical features. For instance, Castro R et al. (28) conducted a comparative analysis of tpp47-PCR and polA-PCR, reporting sensitivities of 75.8% and 69.7%, and specificities of 86.8% and 92.3%, respectively. These findings indicate that different target genes offer distinct advantages in terms of diagnostic performance. Furthermore, a systematic review has indicated that PCR demonstrated a sensitivity ranging from 40% to 70% and a specificity ranging from 60% to 100% in the definitive diagnosis of neurosyphilis (29). In addition, Vanhaecke C et al. (30) employed nested PCR to detect *Treponema pallidum* DNA in the CSF of patients diagnosed with neurosyphilis. The method demonstrated a positive detection rate of 77% among confirmed cases and a specificity of 97%, further supporting the utility of PCR as an auxiliary diagnostic tool in complex clinical scenarios. However, the sensitivity of PCR varies considerably across studies, which may be attributed to factors such as sample preservation methods, disease stage, and HIV co-infection status. Nevertheless, PCR technology continues to provide reliable molecular evidence for the diagnosis of early neurosyphilis and atypical clinical presentations. Its advantages of rapid detection and high specificity are becoming increasingly significant, particularly in resource-limited clinical settings. It is important to note that recent studies have made significant progress in the development of non-invasive diagnostic methods. Xie et al. (31) demonstrated that serum ubiquitin C-terminal hydrolase L1, glial fibrillary acidic protein, and neurofilament light chain may serve as potential biomarkers for neurosyphilis, offering the prospect of avoiding the risks and discomfort associated with lumbar puncture. Chen et al. (32) further validated the diagnostic utility of these biomarkers specifically in HIV-negative neurosyphilis patients, thereby providing a novel and promising tool for clinical diagnosis. With the development of bioinformatics technology, emerging studies have begun to explore the application of multi-omics, artificial intelligence and machine learning in the diagnosis of neurosyphilis. Liu LL et al. (33) identified new potential biomarkers through metabolomics analysis, opening new avenues for molecular diagnosis of neurosyphilis. Li J et al. (34) used proteomics combined with machine learning model technology to identify three biomarkers (SEMA7A, SERPINA3, and ITIH4) and validate their important roles in the diagnosis of neurosyphilis. Furthermore, Zou H et al. (35) developed and validated that a machine learning model with a unified set of predictors simplifies the complex clinical diagnosis of neurosyphilis. By integrating multiparametric data, these techniques are expected to improve the accuracy and timeliness of diagnosis. However, this field is still in its infancy and more confirmatory studies are needed.

### Global differences in epidemiological trends and the importance of global collaboration

This study highlights substantial global variations in the epidemiology of neurosyphilis. Although the volume of research output in this field is comparable between the United States and China, notable differences exist in their epidemiological profiles. Research in the United States predominantly focuses on populations co-infected with HIV, whereas studies in China encompass a broader range of clinical presentations. These distinctions reflect the differing epidemiological contexts and public health approaches adopted in the two countries. The global incidence of neurosyphilis is on the rise, which may be partially attributed to the resurgence of the syphilis epidemic (36). Hook EW et al. pointed out that delayed diagnosis and inappropriate treatment of early syphilis are important factors contributing to the increased incidence of neurosyphilis (37, 38). Epidemiological data indicated that the incidence of primary and secondary syphilis increased by 711% and 174%, respectively, among both women and men in the United States from 2011 to 2021 (36). The prevalence of neurosyphilis among HIV-infected individuals was 1.7% (39). The onset of HIV infection was associated with an increase in the number of reported neurosyphilis cases, particularly those in the early stage. This trend is especially evident among men who have sex with men, highlighting the critical importance of prevention and screening initiatives within high-risk populations. In addition, the interaction between HIV infection and neurosyphilis has also attracted much attention. Studies have found that HIV-Infected individuals have a significantly increased risk of neurosyphilis, and the clinical manifestations may be more complex (40). CD4 counts less than 350/μL and serologic TRUST titers ≥ 1:16 were significant predictors of neurosyphilis in HIV-Positive individuals (41). These findings provide an important basis for risk assessment of neurosyphilis in different populations. Mechanistic insights into HIV co-infection and treponemal neuro-invasiveness. In the face of the continuous rise of global incidence, Wang Qianqiu and other scholars formally proposed the CARE-NS strategy through long-term multi-center collaboration, which provided an operational multidisciplinary alliance framework to bridge the above differences (42). The framework consists of six core modules: coordinated multidisciplinary care (C), alleviating neurological sequelae (A), risk factors & epidemiology (R), etiology & pathogenesis (E), novel diagnostic indicators (N), and social-cost evaluation (S). The framework integrates data sharing, diagnostic standardization, and health economic evaluation into a roadmap. On the one hand, countries can connect corresponding modules under the CARE-NS framework according to their own epidemiological characteristics. For example, the United States strengthens the “R” module of HIV co-infection, and China extends the “E” module of rare phenotypes. On the other hand, through unified data standards and biobank protocols, cross-regional comparison and validation can be realized, so as to accelerate the process from discovery to clinical translation of new markers, and ultimately form an accurate, efficient and sustainable prevention and control system for neurosyphilis on a global scale.

The United States and China made the most prominent contributions, demonstrating their leadership in this field of research. Together, these two countries account for more than 54% of total production, far outnumbering Brazil, which ranks third, and other countries. Beyond China and the United States, countries such as Brazil, the United Kingdom, India, and France have demonstrated unique areas of expertise. UK research has maintained a consistent focus on specific populations, particularly individuals with HIV. Research acknowledges that HIV co-infection alters syphilis progression and clinical presentation, increasing neurosyphilis risk (43, 44). Consequently, more proactive diagnostic and treatment strategies are recommended for these populations. As a region endemic to multiple tropical infectious diseases, Brazil’s research naturally examines the interactions between neurosyphilis and other local infections. For instance, studies list neurosyphilis as a key differential diagnosis for chronic meningitis, alongside tuberculosis and cryptococcosis. Additionally, investigations exploring neurosyphilis as a potential cause of ischemic stroke demonstrate clinical reasoning for comprehensive diagnosis within complex medical contexts (45, 46). A major highlight of French research lies in its innovative diagnostic approaches. Prospective studies in France have evaluated the diagnostic value of nested PCR for detecting Treponema pallidum in cerebrospinal fluid (30). Even more innovatively, French researchers pioneered the detection of Treponema pallidum in tears to diagnose early neurosyphilis (47). If proven effective, this non-invasive sampling method could significantly streamline the diagnostic process, reduce patient discomfort and risks, demonstrating the originality of French scientific thinking and its patient-centered clinical care. Case reports play an important role in neurosyphilis research by bridging clinical experience and academic investigation through the documentation of exceptional cases. Due to the variable clinical symptoms of neurosyphilis, misdiagnosis or underdiagnosis is common. Indian scholars have contributed numerous case reports highlighting the disease’s diverse manifestations. For example, a 20-year compilation of 143 neurosyphilis patients in an Indian hospital identified hallucinations, delusions, and catatonia as the most frequent psychiatric symptoms (48). Other patients presented with atypical clinical signs complicated by multiple comorbidities (49, 50). We also note the research achievements of Italian scholars in neurosyphilis treatment. The enhanced antibiotic regimen proposed by Drago F et al. (51) for early and late syphilis indirectly reduces the subsequent risk of neurosyphilis by increasing the initial cure rate; its potential preventive value has been cited in several reviews. This limited pattern of global collaboration suggests that current research still relies heavily on regional academic resources and study cohorts. This dependence may partly limit the diversity of research perspectives, methodological innovations, and generalizations of findings. Therefore, international collaboration is particularly important, especially by aligning high-level research with the actual needs of regions with high disease burden, which is essential to increase the depth and global impact of research within the field.

### Trends in molecular mechanism research

In this study, the BioBERT biomedical language model was used to conduct in-depth analysis of a large corpus of literature abstracts, and to quantitatively identify core gene targets for molecular mechanism research of neurosyphilis from the perspective of computational linguistics. The spectrum of molecular markers revealed by the analysis reflects the complexity of neurosyphilis pathogenesis. CD4, as the most frequent molecular marker, suggests the central role of T cell immunity in the pathogenesis of neurosyphilis. This is mainly related to two key contexts. First of all, HIV co-infection with syphilis is the most important risk factor for neurosyphilis. CD4^+^ T cell count is the core indicator to evaluate the immune status of HIV-Infected patients, and its level directly affects the host’s ability to fight *Treponema pallidum* (52, 53). Second, CD4^+^ T cells are key effector cells that activate macrophages, drive immune inflammatory responses in the central nervous system, and cause nervous system damage (54). Exploring the infiltration characteristics of CD4^+^ T cells in the disease is helpful to reveal the molecular mechanism of immune activation and the way that *Treponema pallidum* achieves immune evasion and establish chronic infection. The persistently elevated expression of CXCL13 not only reflects the chemotactic activity of B cells within the central nervous system but also has the potential to serve as a biomarker for monitoring disease activity (9). Analysis of the cytokine network revealed a dysregulation in the Th1/Th2 immune balance. The elevated levels of proinflammatory cytokines, including IL-6, IL-1β, and TNF-α, indicate a persistent inflammatory response within the central nervous system of patients with neurosyphilis (55, 56). This inflammatory environment may contribute not only to neuronal damage but also to the underlying mechanisms of cognitive dysfunction. Notably, the concurrent presence of anti-inflammatory cytokines such as IL-10 suggests that the body is attempting to modulate the excessive inflammatory activity (55). In addition to host immune responses, the ability of *Treponema pallidum* to invade the central nervous system is intrinsically linked to its unique structural and genetic characteristics. Studies have shown that *Treponema pallidum* can adhere to and traverse the blood-brain barrier without overtly disrupting its integrity, potentially via receptor-mediated endocytosis and interactions with tight junction proteins (57). Key adhesins such as Tp0751 and Tp0965 have been implicated in modulating endothelial permeability and promoting translocation across cerebral microvascular endothelial cells (58). Furthermore, *Treponema pallidum* has been shown to evade immune detection through antigenic variation, particularly via the TprK gene, which may contribute to persistent central nervous system infection (59). Notably, certain strain genotypes-such as those classified under the 14d/f subtype using enhanced CDC typing-have been associated with a higher likelihood of neurosyphilis (60, 61), suggesting a potential link between microbial genetic background and neuroinvasive potential. Although current evidence is limited by small sample sizes and inconsistent inclusion criteria, these findings underscore the importance of considering both host and pathogen factors in understanding neurosyphilis pathogenesis.

### The matching degree between research hotspots and clinical needs

Keyword cluster analysis revealed the correspondence between current research hotspots and clinical needs. The appearance of “ocular syphilis” and “general paresis” as independent clusters reflects the attention of clinicians to these special manifestations. Minter DJ emphasized that the increase in atypical neurosyphilis manifestations requires clinicians to maintain a high degree of suspicion (62), especially when cognitive dysfunction is present in younger patients. In addition, the prominent position of “case report” clustering also exposed the lack of evidence of evidence-based medicine. Current treatment guidelines for neurosyphilis are mainly based on expert consensus and small case series, lacking the support of large-scale randomized controlled trials. This weakness of the evidence base limits the application of precision medicine in the diagnosis and treatment of neurosyphilis. The results of keyword outbreak analysis showed that “cerebrospinal fluid abnormality”, “treatment”, “benzathine penicillin” and other emerging keywords from 2010 to 2014 were featured, indicating that penicillin-based treatment programs were focused in this stage. Although penicillin is still the drug of choice for the treatment of neurosyphilis, treatment failure and recurrence still occur. In the last decade, several studies have also been devoted to finding new treatment options for syphilis. Although the overall efficacy of ceftriaxone in neurosyphilis has been shown to be comparable to that of benzylpenicillin (52% vs 33%), there is still room for improvement in cure rates (63). Drago F et al. (51) proposed the concept of “enhanced antibiotic regimen” in 2016, adding doxycycline and ceftriaxone on the basis of standard penicillin G. This study took advantage of its high lipid solubility and brain tissue penetration to form “sequency-synergistic” killing, which has been proved to achieve 100% serological cure rate for early and late syphilis. The ability of a drug to penetrate the blood-brain barrier is a key factor in treatment success (64). In the future, more cutting-edge exploration may focus on nanocarriers and targeted delivery. Animal experiments showed that penicillin G encapsulated by polylactic acid-glycolic acid nanoparticles increased the AUC of brain tissue by 6.8 times (65, 66). Outer-membrane vesicles loaded with ceftriaxone and magnetically driven across the blood-brain barrier could kill ceftriaxone-resistant Escherichia coli strains and thus be used for sonodynamic therapy of bacterial meningitis (67). Although these technologies have not yet reached the clinic, Drago F’s enhanced antibiotic regimen provides an immediately scalable transition strategy that provides the drug-dose cornerstone for the next phase of global multicenter randomized controlled trials (the “N” module in the CARE-NS framework). Future studies with larger sample sizes are urgently needed to optimize dosage, course of treatment, and drug combinations to effectively reduce treatment failure and its resulting complications such as neurosyphilis.

### Study limitations and future directions

It is important to acknowledge several limitations of this study. First, as a bibliometric analysis, data collection and processing heavily depend on the software used, and while this approach enables comprehensive analysis of large datasets, it cannot fully replace systematic literature reviews. Second, this study is limited to English articles to ensure database consistency and reproducibility. The restriction to English-language literature may risk overlooking high-quality non-English research. However, given that the vast majority of high-impact studies led by Chinese researchers are published in English, this limitation is unlikely to significantly affect the core conclusions of this analysis. Future inclusion of multilingual literature may further reveal the complete spectrum of research characteristics and evidence-based evidence in different countries. Third, the citation delay effect may lead to underestimating the impact of recent high-quality studies. Despite these limitations, this study provides valuable academic insights into the trends, hotspots, and emerging frontiers in neurosyphilis research.

Future research should focus on the following directions: First, the establishment of standardized diagnostic criteria for neurosyphilis, particularly their applicability in populations co-infected with HIV, requires further validation. Second, large-scale, multicenter studies should be conducted to confirm the clinical utility of novel biomarkers. Third, basic research should be strengthened to elucidate the molecular mechanisms underlying neurosyphilis and provide a theoretical basis for targeted therapeutic strategies. Finally, artificial intelligence and machine learning techniques should be utilized to develop a multi-parameter diagnostic model aimed at improving both the accuracy and timeliness of diagnosis.

## Conclusion

The field of neurosyphilis research is advancing rapidly, marked by significant progress in diagnostic technologies, epidemiological surveillance, and the understanding of molecular mechanisms. However, given the increasingly severe global epidemiological situation, there is a pressing need to enhance international collaboration, establish standardized protocols for diagnosis and treatment, and further explore novel diagnostic markers and therapeutic targets. By integrating multidisciplinary resources, it is anticipated that there will be a substantial improvement in the diagnosis and management of neurosyphilis over the next decade. In particular, the development of non-invasive diagnostic techniques, the validation of emerging biomarkers, and the application of precision medicine strategies are expected to bring transformative advancements to early diagnosis and individualized treatment approaches for neurosyphilis.

## Data Availability

The original contributions presented in the study are included in the article/supplementary material. Further inquiries can be directed to the corresponding author.
